# The contribution of cannabis use to variation in the incidence of psychotic disorder across Europe (EU-GEI): a multicentre case-control study

**DOI:** 10.1016/S2215-0366(19)30048-3

**Published:** 2019-05

**Authors:** Marta Di Forti, Diego Quattrone, Tom P Freeman, Giada Tripoli, Charlotte Gayer-Anderson, Harriet Quigley, Victoria Rodriguez, Hannah E Jongsma, Laura Ferraro, Caterina La Cascia, Daniele La Barbera, Ilaria Tarricone, Domenico Berardi, Andrei Szöke, Celso Arango, Andrea Tortelli, Eva Velthorst, Miguel Bernardo, Cristina Marta Del-Ben, Paulo Rossi Menezes, Jean-Paul Selten, Peter B Jones, James B Kirkbride, Bart PF Rutten, Lieuwe de Haan, Pak C Sham, Jim van Os, Cathryn M Lewis, Michael Lynskey, Craig Morgan, Robin M Murray, EU-GEI WP2 Group, Silvia Amoretti, Manuel Arrojo, Grégoire Baudin, Stephanie Beards, Miquel Bernardo, Julio Bobes, Chiara Bonetto, Bibiana Cabrera, Angel Carracedo, Thomas Charpeaud, Javier Costas, Doriana Cristofalo, Pedro Cuadrado, Covadonga M Díaz-Caneja, Aziz Ferchiou, Nathalie Franke, Flora Frijda, Enrique García Bernardo, Paz Garcia-Portilla, Emiliano González, Kathryn Hubbard, Stéphane Jamain, Estela Jiménez-López, Marion Leboyer, Gonzalo López Montoya, Esther Lorente-Rovira, Camila Marcelino Loureiro, Giovanna Marrazzo, Covadonga Martínez, Mario Matteis, Elles Messchaart, Ma Dolores Moltó, Juan Nacher, Ma Soledad Olmeda, Mara Parellada, Javier González Peñas, Baptiste Pignon, Marta Rapado, Jean-Romain Richard, José Juan Rodríguez Solano, Laura Roldán Díaz, Mirella Ruggeri, Pilar A. Sáiz, Emilio Sánchez, Julio Sanjuán, Crocettarachele Sartorio, Franck Schürhoff, Fabio Seminerio, Rosana Shuhama, Lucia Sideli, Simona A Stilo, Fabian Termorshuizen, Sarah Tosato, Anne-Marie Tronche, Daniella van Dam, Elsje van der Ven

**Affiliations:** a Social, Genetic and Developmental Psychiatry Centre, Institute of Psychiatry, King's College London, London, UK; b Department of Addiction, Institute of Psychiatry, King's College London, London, UK; c Institute of Psychiatry, Psychology and Neuroscience and Department of Psychosis Studies, Institute of Psychiatry, King's College London, London, UK; d Department of Health Service and Population Research, Institute of Psychiatry, King's College London, London, UK; e National Institute for Health Research (NIHR) Mental Health Biomedical Research Centre at South London and Maudsley NHS Foundation Trust and King's College London, UK; f South London and Maudsley NHS Mental Health Foundation Trust, London, UK; g Addiction and Mental Health Group (AIM), Department of Psychology, University of Bath, Bath, UK; h Department of Psychiatry, University of Cambridge, Cambridge, UK; i Department of Experimental Biomedicine and Clinical Neuroscience, University of Palermo, Palermo, Italy; j Department of Medical and Surgical Science, Psychiatry Unit, Alma Mater Studiorum Università di Bologna, Bologna, Italy; k INSERM U955, Equipe 15, Institut National de la Santé et de la Recherche Médicale, Créteil, Paris, France; l Department of Child and Adolescent Psychiatry, Hospital General Universitario Gregorio Marañón, School of Medicine, Universidad Complutense, IiSGM (CIBERSAM), Madrid, Spain; m Etablissement Public de Santé Maison Blanche, Paris, France; n Department of Psychiatry, Early Psychosis Section, Academic Medical Centre, University of Amsterdam, Amsterdam, Netherlands; o Barcelona Clinic Schizophrenia Unit, Neuroscience Institute, Hospital clinic, Department of Medicine, University of Barcelona, IDIBAPS, CIBERSAM, Barcelona, Spain; p Division of Psychiatry, Department of Neuroscience and Behaviour, Ribeirão Preto Medical School, University of São Paulo, São Paulo, Brazil; q Department of Preventative Medicine, Faculdade de Medicina FMUSP, University of São Paulo, São Paulo, Brazil; r Rivierduinen Institute for Mental Health Care, Leiden, Netherlands; s CAMEO Early Intervention Service, Cambridgeshire & Peterborough NHS Foundation Trust, Cambridge, UK; t Psylife Group, Division of Psychiatry, University College London, London, UK; u Department of Psychiatry and Neuropsychology, School for Mental Health and Neuroscience, South Limburg Mental Health Research and Teaching Network, Maastricht University Medical Centre, Maastricht, Netherlands; v Centre for Genomic Sciences, Li KaShing Faculty of Medicine, The University of Hong Kong, Hong Kong, China; w Brain Centre Rudolf Magnus, Utrecht University Medical Centre, Utrecht, The Netherlands

## Abstract

**Background:**

Cannabis use is associated with increased risk of later psychotic disorder but whether it affects incidence of the disorder remains unclear. We aimed to identify patterns of cannabis use with the strongest effect on odds of psychotic disorder across Europe and explore whether differences in such patterns contribute to variations in the incidence rates of psychotic disorder.

**Methods:**

We included patients aged 18–64 years who presented to psychiatric services in 11 sites across Europe and Brazil with first-episode psychosis and recruited controls representative of the local populations. We applied adjusted logistic regression models to the data to estimate which patterns of cannabis use carried the highest odds for psychotic disorder. Using Europe-wide and national data on the expected concentration of Δ^9^-tetrahydrocannabinol (THC) in the different types of cannabis available across the sites, we divided the types of cannabis used by participants into two categories: low potency (THC <10%) and high potency (THC ≥10%). Assuming causality, we calculated the population attributable fractions (PAFs) for the patterns of cannabis use associated with the highest odds of psychosis and the correlation between such patterns and the incidence rates for psychotic disorder across the study sites.

**Findings:**

Between May 1, 2010, and April 1, 2015, we obtained data from 901 patients with first-episode psychosis across 11 sites and 1237 population controls from those same sites. Daily cannabis use was associated with increased odds of psychotic disorder compared with never users (adjusted odds ratio [OR] 3·2, 95% CI 2·2–4·1), increasing to nearly five-times increased odds for daily use of high-potency types of cannabis (4·8, 2·5–6·3). The PAFs calculated indicated that if high-potency cannabis were no longer available, 12·2% (95% CI 3·0–16·1) of cases of first-episode psychosis could be prevented across the 11 sites, rising to 30·3% (15·2–40·0) in London and 50·3% (27·4–66·0) in Amsterdam. The adjusted incident rates for psychotic disorder were positively correlated with the prevalence in controls across the 11 sites of use of high-potency cannabis (r = 0·7; p=0·0286) and daily use (r = 0·8; p=0·0109).

**Interpretation:**

Differences in frequency of daily cannabis use and in use of high-potency cannabis contributed to the striking variation in the incidence of psychotic disorder across the 11 studied sites. Given the increasing availability of high-potency cannabis, this has important implications for public health.

**Funding source:**

Medical Research Council, the European Community's Seventh Framework Program grant, São Paulo Research Foundation, National Institute for Health Research (NIHR) Biomedical Research Centre (BRC) at South London and Maudsley NHS Foundation Trust and King's College London and the NIHR BRC at University College London, Wellcome Trust.

## Introduction

Many countries have legalised or decriminalised cannabis use, leading to concerns that this might result in an increase in cannabis use and associated harm,^1^, ^2^ even if the latter only affects a minority of the population.^3^ Cross-sectional and prospective epidemiological studies^4^, ^5^ as well as biological evidence^6^ support a causal link between cannabis use and psychotic disorder. Meta-analysis shows a dose–response association with the highest odds of psychotic disorder in those with the heaviest cannabis use.^7^ Nevertheless, it is not clear whether, at a population level, patterns of cannabis use influence rates of psychotic disorder.^8^, ^9^, ^10^

A systematic review^11^ has described a five-times variation in the incidence of schizophrenia worldwide. A transnational case-control study (EU-GEI) has reported an eight-times difference in the incidence of psychotic disorder across 16 European sites plus one in Brazil.^12^ Differences in the distribution of risk factors for psychosis, such as cannabis use, among the populations studied might contribute to these variations.

**Research in context**

**Evidence before this study**

The evidence reporting the dose-dependent association between cannabis use and psychotic disorders has been summarised in the meta-analysis by Marconi and colleagues. We searched PubMed for studies published up to March 31, 2018, that had specifically measured the impact of high-potency cannabis use on the odds of psychotic disorder (not psychotic symptoms or psychosis in general) or that had calculated the proportion of new cases of psychotic disorder arising in specific populations that were attributable to the use of high-potency cannabis, using the terms “psychotic disorders” and “high potency cannabis” or “skunk-super skunk” or “high THC cannabis”; we also included the term “population attributable fraction”. Finally, we searched for studies that reported the impact of any use of cannabis on the incidence of psychotic disorder or schizophrenia. Three studies met our inclusion criteria. Boydell and colleagues speculated that an increase in the incidence rates of schizophrenia between 1965 and 1999 in south London might be related to the increase, over the same period, in the prevalence of cannabis use in the year before first presentation. Our two previous case-control studies showed that high-potency cannabis, especially when used daily, carries the highest risk for psychotic disorder and that, assuming causality, 24% of new cases of psychotic disorder in south London could be attributed to the use of high potency cannabis.

**Added value of this study**

This multicentre case-control study across ten European and one Brazilian site replicates the strong effect of daily use of high-potency cannabis on the odds for psychotic disorder in the whole sample—which, to our knowledge, is the largest to date to address this question. This effect was particularly visible in London and Amsterdam. Additionally, we show that, assuming causality, if high-potency cannabis types were no longer available, then 12% of cases of first-episode psychosis could be prevented across Europe, rising to 30% in London and 50% in Amsterdam. Most importantly, we provide the first direct evidence that cannabis use has an effect on variation in the incidence of psychotic disorders. We show that differences in the prevalence of daily use of cannabis, and in use of high-potency cannabis, among the controls from the different study sites made a major contribution to the striking variations in the incidence rates of psychotic disorder that we have previously reported across the same sites.

**Implications of all available evidence**

In the context of the well reviewed epidemiological and biological evidence of a causal link between heavy cannabis use and psychotic disorders, our findings have substantial implications for mental health services and public health. Education is needed to inform the public about the mental health hazards of regular use of high-potency cannabis, which is becoming increasingly available worldwide.

Therefore, using data from the EU-GEI case-control study of first-episode psychosis and the previously published data on incidence,^12^ we sought to describe differences in patterns of cannabis use across sites, identify the measure of cannabis use with the strongest impact on odds of psychotic disorder across sites, calculate the population attributable fraction (PAF) for the patterns of cannabis use associated with the highest odds for psychosis, and test whether differences in patterns of cannabis use contribute to variations in the incidence of psychotic disorder across sites.

## Methods

### Study design

The EU-GEI project set out to estimate the incidence of psychosis and recruit first-episode psychosis cases and controls to investigate risk factors for psychotic disorder. First, incidence rates were estimated^12^ by identifying all individuals with a first episode of psychosis who presented to mental health services between May 1, 2010, and April 1, 2015, in 17 areas in England, France, the Netherlands, Italy, Spain, and Brazil (appendix). Second, to investigate risk factors, we attempted to assess 1000 first-episode cases and 1000 population-based controls during the same period.

### Participants

Patients presenting with their first episode of psychosis were identified by trained researchers who carried out regular checks across the mental health services within the 17 catchment areas (one site per catchment area). Patients were eligible if they were aged 18–64 years and resident within the study areas at the time of their first presentation with a diagnosis of psychosis by ICD-10 criteria (F20–33); details are provided in the supplementary methods and in previous publications.^12^ Cases were approached via their clinical team and invited to participate. Using the Operational Criteria Checklist algorithm, all cases interviewed received a research-based diagnosis.^13^ Patients were excluded if they had been previously treated for psychosis or if they met criteria for organic psychosis (F09) or for psychotic symptoms resulting from acute intoxication (F1X.5).

We adopted quota sampling strategies to guide the recruitment of controls. Accurate local demographic data were used to set quotas for controls to ensure the samples' representativeness of each catchment area's population at risk in terms of age, gender, and ethnicity. Potential controls were initially identified on the basis of locally available sampling strategies, most commonly random sampling from lists of all postal addresses and from general practitioner lists from randomly selected surgeries. To achieve representation of hard-to-reach groups (eg, young men), we then tried to oversample them using more ad-hoc approaches such as internet and newspaper advertisements, and leaflets at local stations, shops, and job centres. Controls were excluded if they had received a diagnosis of, or treatment for, psychotic disorder.

All participants provided informed, written consent. Ethical approval was provided by research ethics committees in each site.

### Measures

We obtained sociodemographic data using the Medical Research Council Sociodemographic Schedule, as described previously.^14^ An updated version of the modified Cannabis Experience Questionnaire^15^ (CEQ_EU-GEI_) was used to gather detailed history of use of cannabis and other recreational drugs (appendix). To minimise recall bias, none of the recruitment materials for cases or controls mentioned cannabis or referred to its potential role as risk factor for psychotic disorder. Participants were asked if they had ever used cannabis in their lifetime; if the answer was yes, they were then asked to give details on their pattern of use. Questions on the type of cannabis used made no reference to its potency and allowed participants to report the colloquial name, in any language, of the cannabis they used.

We included six measures of cannabis use in the initial analyses, including lifetime cannabis use (ie, whether or not the individual had ever used cannabis), currently using cannabis, age at first use of cannabis,^16^ lifetime frequency of use (ie, the frequency that characterised the individual's most consistent pattern of use), and money spent weekly on cannabis during their most consistent pattern of use. Using data published in the European Monitoring Centre for Drugs and Drug Addiction 2016 report^17^ that reported the concentration of Δ^9^-tetrahydrocannabinol (THC) in the types of cannabis available across Europe, supplemented by national data for each included country,^18^, ^19^, ^20^, ^21^, ^22^, ^23^, ^24^, ^25^, ^26^ we created the final measure of cannabis potency (appendix).

### Statistical analysis

We used complete case analyses for all analyses using Stata version 14. We used inverse probability weights to account for any oversampling of controls relative to the populations at risk (appendix); we gave each control's data a weight inversely proportional to their probability of selection given their key demographics (age, gender, and ethnicity) using census data on relevant populations. These weights were applied in all analyses.

To identify potential confounders, we used χ^2^ and *t* tests to test for an association between sociodemographic data and the data on drug use with case-control status in the whole sample. On the basis of the χ^2^ and t tests, data on the use of other recreational drugs were included as confounders in the main analyses, with low or no use scored as 0 and use scored as 1 in categorical variables: tobacco (never used or smoked <10 cigarettes per day *vs* smoked ≥10 cigarettes or more per day); stimulants, hallucinogens, ketamine, and novel psychoactive substances (so-called legal highs; never tried *vs* ever tried); and mean number of alcoholic drinks consumed daily on an average week. All sociodemographic and drug-use variables associated with case-control status were controlled for in all analyses (appendix).

We applied adjusted logistic regression models to estimate the effect of each of the six measures of cannabis use on the odds of a psychotic disorder (ie, case status). The data have a multilevel structure because cases and controls are nested within sites. To take account of this clustering in the logistic regression analysis, we used the cluster option in Stata. We fitted interaction terms to logistic models. These interaction models, using likelihood ratio tests, were run to investigate whether individual measures of cannabis use interacted with each other to significantly increase the odds ratios (ORs) for psychotic disorder and whether the ORs for psychotic disorder of the individual measures of cannabis use varied significantly by site.

The STATA *punafcc* command was used to calculate the population attributable fraction (PAF) with 95% CIs for the two cannabis use measures that carried the largest adjusted OR for psychosis. The PAF measures the population effect of an exposure by providing an estimate of the proportion of disorder that would be prevented if the exposure were removed, assuming causality.

To account for potential selection bias, we did a probabilistic sensitivity analysis using the STATA *episensi* command.^27^ This analysis assumes that we can assign prior probability distributions for the bias parameters, which capture the uncertainty about those parameters, and use these distributions in a probabilistic sensitivity analysis (appendix).

Finally, we used Pearson's correlation to test for an association between the incidence rates for psychotic disorder adjusted for ethnic minority status in each site and the prevalence of daily cannabis use and use of high-potency cannabis in the controls as representing the general population for each site.

### Role of the funding source

Study funders contributed to the salaries of the research workers employed but did not participate in the study design, data analyses, data interpretation, or writing of the manuscript. All authors had full access to the study data and had final responsibility for the decision to submit for publication.

## Results

Between May 1, 2010, and April 1, 2015, we approached 1519 patients with first-episode psychosis; 356 (23%) refused to participate, 19 (1%) could not consent because of language barriers, and 14 (0·9%) were excluded because they did not meet the age inclusion criteria. Patients who refused to participate were older (p=0·0015), more likely to be women (p=0·0063) and of white European origin (p<0·0001; appendix).

Thus, 1130 cases took part. These cases were broadly representative for gender and ethnicity of the incidence sample, although younger (mean age 31·2 years [SD 10·6], median 29 years [IQR 23–37] for cases *vs* mean 34·5 years [12·0], median 31 years [23·0–41·0] for the total incidence; p<0·0001; details by site are available in the appendix). All 17 sites contributed to the recruitment of 1499 population controls except for Maison Blanche, which was consequently excluded from the analysis (appendix).

Most sites had minimal missing sociodemographic (≤3%) or CEQ_EU-GEI_ data (<5%). However, Verona, Santiago, Oviedo, Valencia, and Cuenca had at least 10% of data missing on the measures of cannabis use or on one or more of the main confounding variables; therefore, given their small sample sizes there was insufficient data to include these sites in the analysis. This resulted in 901 cases and 1237 controls for analysis.

Compared with controls, cases were younger, more often men, and from ethnic minorities, than the controls (table 1). Controls were more likely to have pursued higher education (p<0·0001) and to have been employed a year before assessment than cases (p<0·0001; table 1); the differences in gender, ethnicity, education, and employment are those expected when comparing patients with psychosis with general population samples.

**Table 1:** Sociodemographics and lifetime history of substance misuse across all included cases and controls

		**Controls (n=1237)**	**Cases (n=901)**	**p value**
Age, years		36·0 (12·8)	31·2 (10·6)	<0·0001
Gender		..	..	<0·0001
	Female	655 (53·0%)	343 (38·1%)	..
	Male	582 (47·0%)	558 (61·9%)	..
Self-reported ethnicity		..	..	<0·0001
	White	930 (75·2%)	532 (59·0%)	..
	Black	118 (9·5%)	168 (18·6%)	..
	Mixed	113 (9·1%)	104 (11·5%)	..
	Asian	33 (2·7%)	32 (3·6%)	..
	North African	23 (1·9%)	42 (4·7%)	..
	Others	20 (1·6%)	23 (2·6%)	..
Education		..	..	<0·0001
	School with no qualifications	66 (5·3%)	158 (17·5%)	..
	School qualifications	159 (12·9%)	232 (25·7%)	..
	Vocational or undergraduate	826 (66·8%)	465 (51·6%)	..
	Postgraduate	177 (14·3%)	36 (4·0%)	..
	Data missing	9 (0·7%)	10 (1·1%)	..
Employment status 1 year before assessment		..	..	<0·0001
	Unemployed	95 (7·7%)	169 (18·8%)	..
	Economically inactive (ie, house person)	122 (9·9%)	62 (6·9%)	..
	Student	215 (17·4%)	146 (16·2%)	..
	Employee (full time/part time/self-employed)	805 (65·1%)	488 (54·2%)	..
	Data missing	0	36 (4·0%)	..
Lifetime cannabis use		..	..	<0·001
	Yes	574 (46·4%)	585 (64·9%)	..
	No	650 (52·5%)	303 (33·6%)	..
	Data missing	13 (1·1%)	13 (1·4%)	..
Lifetime tobacco use		..	..	<0·0001
	Smokes ≥10 cigarettes per day	158 (12·8%)	296 (32·9%)	..
	Smokes <10 cigarettes per day	238 (19·2%)	182 (20·1%)	..
	Never used	838 (67·8%)	421 (46·8%)	..
	Data missing	3 (0·2%)	2 (0·2%)	..
Lifetime use of other drugs				
	Legal highs	30 (2·4%)	39 (4·3%)	0·0142
	Stimulants	149 (12·0%)	196 (21·8%)	<0·0001
	Hallucinogens	111 (9·0%)	131 (14·5%)	<0·0001
	Ketamine	35 (2·8%)	55 (6·1%)	0·0002
	Data missing	2 (0·2%)	0	..

Data are n (%) or mean (SD).

More cases than controls reported having ever used cannabis, having smoked ten tobacco cigarettes or more a day, or having tried other recreational drugs (table 1). We found no difference between cases and controls in the mean number of alcoholic drinks consumed every day on an average week (5·2 drinks [SD 0·4] among controls *vs* 4·8 drinks [0·4] among cases; median 2·0 drinks [IQR 0·0–6·0] for controls *vs* 1·0 drink [0·0–4·0]; p=0·45).

An adjusted logistic regression model showed that those who had ever used cannabis had a modest increase in odds of psychotic disorder compared with those who had never used it (table 2); the odds were slightly greater in those who started to use cannabis at age 15 years or younger.

**Table 2:** Measure of cannabis use and ORs for psychotic disorders for case-control sample across 11 sites

		**Controls (n=1237)**	**Cases (n=901)**	p value*	**Crude OR (95% CI)**†	**p value**	**Fully adjusted OR (95% CI)**†	**p value**
Lifetime cannabis use‡		..	..	<0·0001				
	No	650 (52·5%)	303 (33·6%)	..	1 (ref)	..	1 (ref)	..
	Yes	574 (46·4%)	585 (64·9%)	..	2·45 (2·0–2·9)	<0·0001	1·3 (1·1–1·6)	0·0225
Currently using cannabis		132 (10·7%)	198 (22·0%)	0·00349	2·7 (2·1–3·5)	<0·0001	1·1 (0·9–1·5)	0·36
First used cannabis age ≤15 years old		169 (13·7%)	257 (28·6%)	<0·0001	3·9 (3·0–4·9)	<0·0001	1·6 (1·1–2·1)	0·0122
Lifetime frequency of use		..	..	<0·0001				
	Never or occasional use	1061 (85·8%)	528 (58·7%)	..	1 (ref)	..	1 (ref)	..
	Used more than once a week	92 (7·4%)	107 (11·9%)	..	2·5 (1·9–3·5)	<0·0001	1·4 (1·0–2·0)	0·066
	Daily use	84 (6·8%)	266 (29·5%)	..	6·2 (4·8–8·2)	<0·0001	3·2 (2·2–4·1)	<0·0001
Spent at least €20 per week on cannabis		40 (3·2%)	156 (17·4%)	<0·0001	5·6 (4·0–7·7)	<0·0001	2·5 (1·6–3·8)	<0·0001
Lifetime use of cannabis by potency§		..	..	<0·0001				
	Low potency (THC <10%)	331 (26·7%)	251 (27·9%)	..	2·0 (1·6–2·5)	<0·0001	1·1 (0·9–1·5)	0·38
	High potency (THC ≥10%)	240 (19·4%)	334 (37·1%)	..	3·2 (2·6–4·0)	<0·0001	1·6 (1·2–2·2)	0·0032

Crude ORs are adjusted only for age, gender, and ethnicity whereas fully adjusted ORs are additionally adjusted for level of education, employment status, tobacco, stimulants, ketamine, legal highs, and hallucinogenics. OR=odds ratio. THC=Δ^9^-tetrahydrocannabinol.

* p value for χ^2^ test.

† Reference group for both crude and adjusted ORs is the never users unless specified otherwise.

‡ Data were missing for 13 individuals in each group.

§ Data were missing for three controls.

Daily cannabis use was associated with increased odds of psychotic disorder compared with never having used it (table 2); this remained largely unchanged when taking into account age at first use (OR 3·1, 95% CI 2·1–5·2), money spent (2·9, 1·9–4·4), and type of cannabis used (2·6, 2·0–3·9). Those who spent €20 or more a week showed more than a doubling in the odds of a psychotic disorder (2·5, 1·6–3·8), which dropped to 1·3 (95% CI 1·0–2·1) after controlling for daily use and type of cannabis used; we observed no interaction between daily use and money spent (p=0·67).

Use of high-potency cannabis (THC ≥10%) modestly increased the odds of a psychotic disorder compared with never use (table 2); this remained largely unchanged after controlling for daily use (OR 1·5, 95% CI 1·1–2·6). Those who had started using high-potency cannabis by age 15 years showed a doubling of risk (2·3, 1·4–3·1), without evidence of interaction (p=0·63).

**Figure 1:**
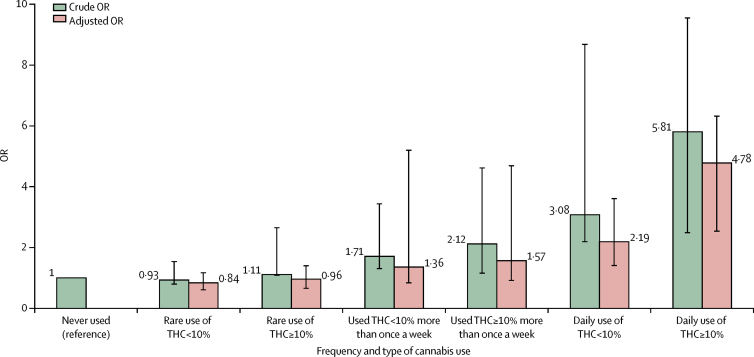
Crude and fully adjusted ORs of psychotic disorders for the combined measure of frequency plus type of cannabis use in the whole sample Crude ORs are adjusted only for age, gender and ethnicity and fully adjusted ORs are additionally adjusted for level of education, employment status, and use of tobacco, stimulants, ketamine, legal highs, and hallucinogenics. Error bars represent 95% CIs. OR=odds ratio.

Frequency of use and type of cannabis used were combined to generate a single-measure of frequency plus type of use because these two measures had the highest ORs. Adjusted logistic regression indicated that daily use of high-potency cannabis carried more than a four-times increase in the risk of psychotic disorder (OR 4·8, 95% CI 2·5–6·3) compared with never having used cannabis; the odds were lower for those who used low-potency cannabis daily (2·2, 1·4–3·6; figure 1). Nevertheless, there was no evidence of interaction between frequency of use and type of cannabis used (p=0·25).

When considering variation by site, neither the ORs for daily use (p=0·25) nor those for high-potency cannabis (p=0·45), compared with never use, varied significantly across sites (table 3). The observed differences in ORs for daily use ranged from 7·1 (95% CI 3·4–11·8) in Amsterdam to 1·1 (0·4–12·2) in Puy de Dôme. Similarly, the differences in the ORs for use of high-potency cannabis, ranging from 3·6 (1·5–7·7) in Amsterdam to 0·6 (0·1–2·5) in Palermo, are consistent with the geographical differences in its availability.^17^

**Table 3:** PAFs for daily use of cannabis and use of high-potency cannabis in the whole sample and by site

	**Fully adjusted OR (95% CI)**	**Prevalence of exposure in controls**	**Prevalence of exposure in cases**	**PAF (95% CI)**
**High-potency cannabis (THC ≥10%)**				
Whole sample	1·6 (1·2–2·2)	19·1%	35·1%	12·2% (3·0–16·1)*
London (UK)	2·4 (1·4–4·0)	26·0%	51·5%	30·3% (15·2–40·0)*
Cambridge (UK)	1·3 (0·4–4·3)	11·0%	34·7%	8·2% (0·5–18·7)
Amsterdam (Netherlands)	3·6 (1·5–7·7)	54·0%	69·6%	50·3% (27·4–66·0)*
Gouda and Voorhout (Netherlands)	1·5 (0·8–3·1)	18·2%	36·0%	12·2% (8·7–25·3)*
Paris (Val-de-Marne; France)	2·1 (0·8–3·6)	21·0%	35·9%	18·9% (14·6–36·0)*
Puy de Dôme (France)	1·5 (0·4–5·8)	3·7%	7·1%	2·3% (0·6–17·2)
Madrid (Spain)	2·0 (0·7–5·7)	15·1%	34·0%	17·2% (0·9–25·0)
Barcelona (Spain)	1·6 (0·5–5·1)	7·8%	13·2%	4·7% (0·5–12·4)
Bologna (Italy)	1·2 (0·8–1·7)	8·7%	11·1%	1·9% (0·6–16·3)
Palermo (Italy)	0·6 (0·1–2·5)	5·2%	4·3%	Not calculated
Ribeirão Preto (Brazil)	2·1 (0·6–11·3)	1·5%	3·6%	1·9% (0·3–4·1)
**Daily cannabis use**				
Whole sample	3·2 (2·2–4·1)	6·8%	29·5%	20·4% (17·6–22·0)*
London (UK)	3·6 (1·4–4·4)	11·7%	29·0%	21·0% (11·1–31·2)*
Cambridge (UK)	2·2 (0·8–6·5)	4·0%	20·2%	10·4% (4·7–21·0)*
Amsterdam (Netherlands)	7·1 (3·4–11·8)	13·1%	51·0%	43·8% (34·0–69·1)*
Gouda and Voorhout (Netherlands)	2·8 (1·4–20·3)	6·0%	27·0%	17·4% (1·1–23·1)*
Paris (Val-de-Marne; France)	2·8 (1·7–12·3)	11·6%	32·3%	20·8% (13·5–36·1)*
Puy de Dôme (France)	1·1 (0·4–12·2)	6·0%	11·0%	1·2% (0·8–15·4)
Madrid (Spain)	2·5 (2·1–7·3)	10·5%	21·2%	12·7% (3·7–14·2)*
Barcelona (Spain)	1·8 (0·8–8·7)	8·3%	18·9%	8·6% (0·6–9·9)
Bologna (Italy)	2·0 (0·5–5·8)	4·1%	17·3%	8·2% (0·8–11·7)
Palermo (Italy)	1·7 (0·7–9·7)	5·1%	17·1%	6·3% (0·9–21·1)
Ribeirão Preto (Brazil)	2·4 (1·5–7·5)	7·4%	25·0%	14·5% (10·2–24·1)*

OR=odds ratio. PAF=population attributable fraction.

* p<0·05.

**Figure 2:**
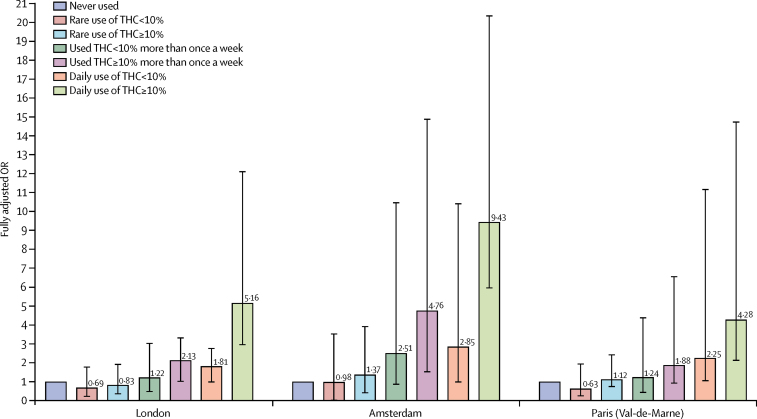
Fully adjusted ORs of psychotic disorders for the combined measure of frequency plus type of cannabis use in three sites Data are shown for the three sites with the greatest consumption of cannabis: London (201 cases, 230 controls), Amsterdam (96 cases, 101 controls), and Paris (54 cases, 100 controls). Error bars represent 95% CIs. OR=odds ratio.

In the three sites with the greatest consumption of high-potency cannabis, daily use of high-potency cannabis was associated with the greatest increase in the odds for psychotic disorder compared with never having used: four times greater in Paris, five times greater in London, and more than nine times greater in Amsterdam (figure 2).

Based on the prevalence of daily cannabis use, and use of high potency cannabis, in cases and controls and the corresponding adjusted ORs, we estimated the PAFs for the whole sample and for each of the sites (table 3). Assuming causality, the proportion of new cases of psychotic disorder in the whole sample attributable to daily use was 20·4% (95% CI 17·6–22·0) and 12·2% (3·0–6·1) for use of high-potency cannabis (table 3).

The PAF analysis revealed variations by sites, ranging from 43·8% (95% CI 34·0–69·1) of new cases of psychotic disorder in Amsterdam being attributable to daily use to just 1·2% (0·8–15·4) of cases in Puy de Dôme. Furthermore, the PAF for use of high-potency cannabis ranged from 50·3% (27·4–66·0) of cases in Amsterdam to 1·9% (0·6–16·3) estimated in Bologna. We did not calculate the PAF for Palermo because there was no main effect of use of high-potency cannabis on the odds for psychotic disorder.

The probabilistic sensitivity analyses we ran suggest that selection bias is unlikely to explain our findings (appendix). After correction for selection bias, the OR for daily cannabis use (5·7, 95% CI 3·5–9·4) was similar to the original OR (5·7, 4·4–7·5). However, the CI for the corrected OR was wider than that for the original OR, suggesting a wider range of possible values for the true OR with 95% certainty. The results of the probabilistic sensitivity analysis to estimate the potential effects of selection bias on high potency cannabis use were similar (appendix).

**Figure 3:**
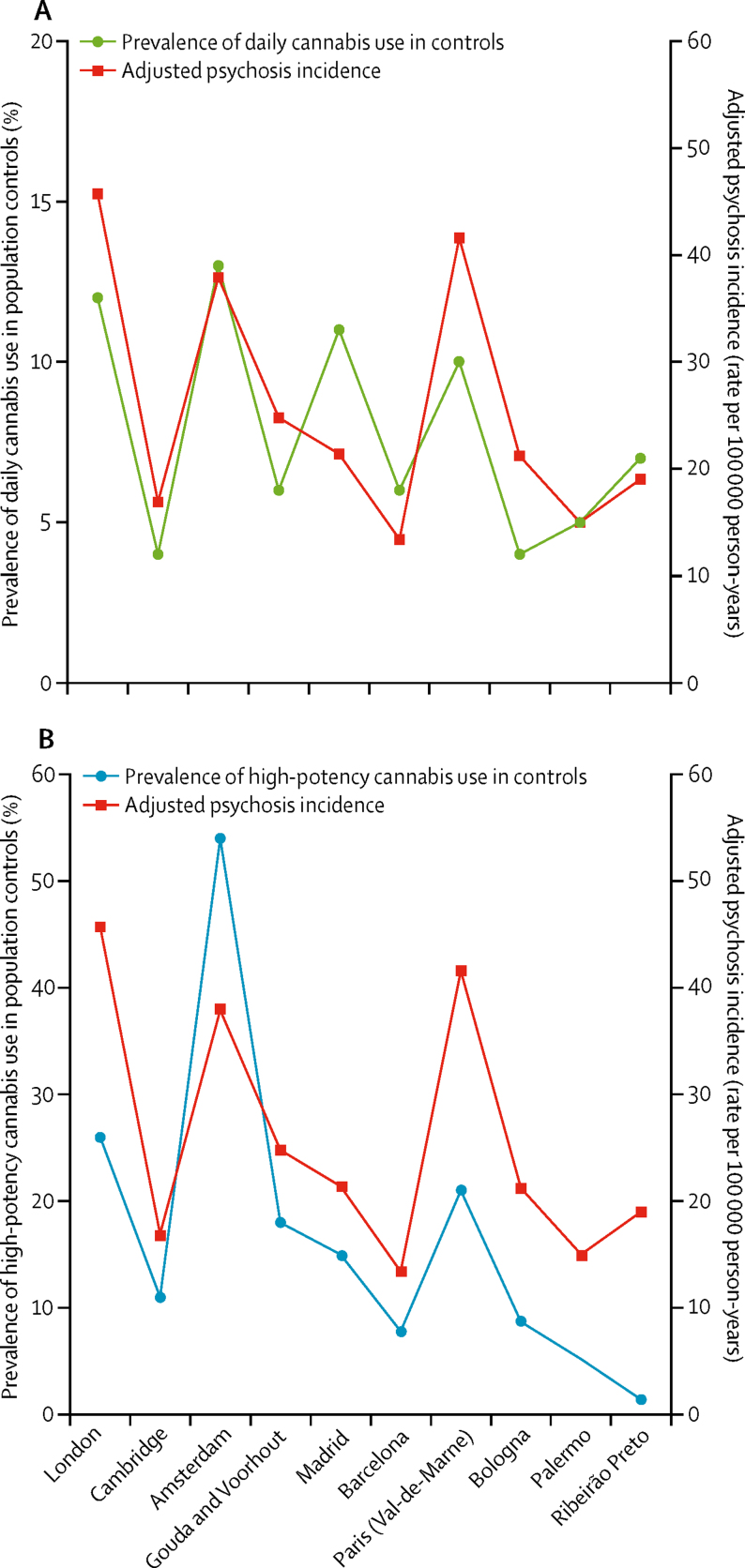
Adjusted incidence rates for all psychosis for the 11 sites plotted against the prevalence of daily use in the population controls (A) and prevalence of use of high-potency cannabis in the population controls (B) Incidence rates are adjusted for age, gender, and ethnicity. Puy-de-Dôme is not included because data on ethnicity were missing for 27 (66%) of 42 incidence cases, therefore the adjusted incidence rate for this site was not calculated.

The EU-GEI incidence study reported an eight-times variation in the incidence rates of psychotic disorder adjusted for age, gender, and ethnic minority status across the study sites.^12^ We found a correlation between the adjusted incidence rates for psychotic disorder in our 11 sites and the prevalence of daily cannabis use in controls (*r*=0·8; p=0·0109). Sites where daily use was common such as London (26 [11·7%] of 223 controls) and Amsterdam (13 [13·0%] of 100 controls) had among the highest adjusted incidence rates (45·7 cases per 100 000 person-years in London and 37·9 per 100 000 person-years in Amsterdam). This differed from sites such as Bologna where daily use was less frequent (three [4·6%] of 65 controls) and the adjusted incidence rate was half that of London (21·0 cases per 100 000 per person years; figure 3).

Similarly, we found a correlation between adjusted incidence rates for psychotic disorder and the prevalence of use of high-potency cannabis in controls across the 11 sites (*r*=0·7; p=0·0286). Amsterdam (54 [54·0%] of 100 controls), London (58 [26·0%] of 223 controls), and Paris (21 [21·0%] of 100 controls) had the highest prevalence of use of high-potency cannabis in controls and the highest adjusted incidence rates for all psychosis (45·7 per 100 000 person-years in London, 37·9 in Amsterdam, and 46·1 in Paris; figure 3). The prevalence of daily use and the prevalence of use of high-potency cannabis in controls were only modestly correlated (*r*=0·2; p=0·0413), therefore we report data for both (figure 3).

## Discussion

Our main findings show that among the measures of cannabis use tested, the strongest independent predictors of whether any given individual would have a psychotic disorder or not were daily use of cannabis and use of high-potency cannabis. The odds of psychotic disorder among daily cannabis users were 3·2 times higher than for never users, whereas the odds among users of high-potency cannabis were 1·6 times higher than for never users. Starting to use cannabis by 15 years of age modestly increased the odds for psychotic disorder but not independently of frequency of use or of the potency of the cannabis used. These measures of extent of exposure did not interact with each other, nor did they interact with the sites. This lack of interaction between degree of cannabis use (ie, daily use of cannabis or use of high-potency cannabis) and site might reflect insufficient power in our study; however, it could also indicate that although the magnitude of the effect might vary depending on the degree of cannabis use, there is a consistent effect of daily use and use of high-potency cannabis on the ORs for psychotic disorders across all study sites.

We replicated our previous finding^28^ that daily use of high-potency cannabis is most strongly associated with case-control status. Compared with never users, participants who used high-potency cannabis daily had four-times higher odds of psychosis in the whole sample, with a five-times increase in London and a nine-times increase in Amsterdam. We also saw that, in the whole sample, daily use of high-potency cannabis was associated with a doubling in the OR for psychotic disorder. The large sample size and the different types of cannabis available across Europe have allowed us to report that the dose–response relationship characterising the association between cannabis use and psychosis^7^ reflects not only the use of high-potency cannabis but also the daily use of types with an amount of THC consistent with more traditional varieties.

Use of high-potency cannabis was a strong predictor of psychotic disorder in Amsterdam, London, and Paris where high-potency cannabis was widely available, by contrast with sites such as Palermo where this type was not yet available. In the Netherlands, the THC content reaches up to 67% in Nederhasj and 22% in Nederwiet; in London, skunk-like cannabis (average THC of 14%) represents 94% of the street market^29^ whereas in countries like Italy, France, and Spain, herbal types of cannabis with THC content of less than 10% were still commonly used.^17^, ^18^

Thus our findings are consistent with previous epidemiological and experimental evidence suggesting that the use of cannabis with a high concentration of THC has more harmful effects on mental health than does use of weaker forms.^28^, ^30^, ^31^

The novelty of this study is its multicentre structure and the availability of incidence rates for psychotic disorder for all the sites. This has allowed us, for the first time, to show how the association between cannabis use and risk of psychosis varies geographically depending on prevailing patterns of use, and how the latter contributes to variation in incidence rates for psychotic disorder.

Variations in patterns of cannabis use across the sites translated into differences in the proportion of new cases of psychotic disorder attributable to cannabis use. We estimated, assuming causality, that 20% of new cases of psychotic disorder across all our sites could have been prevented if daily use of cannabis had been abolished; the PAF for daily use was 21% for London, similar to that previously reported,^3^ but ranged from 44% in Amsterdam to 6% in Palermo. The local availability of high-potency types of cannabis resulted in a PAF of 50% for Amsterdam and 30% for London. Therefore, assuming causality, if high-potency cannabis were no longer accessible, the adjusted incidence rates for all psychotic disorder in Amsterdam would drop from 37·9 to 18·8 cases per 100 000 person-years and in London from 45·7 to 31·9 cases per 100 000 person-years.

Finally, we report what, to our knowledge, is the first evidence that differences in the prevalence of daily use and use of high-potency cannabis in the controls correlate with the variation in the adjusted incidence rates for psychotic disorder across the study sites. Our results show that in areas where daily use and use of high-potency cannabis are more prevalent in the general population, there is an excess of cases of psychotic disorder.

Our findings need to be appraised in the context of limitations. Data on cannabis use are not validated by biological measures, such as urine, blood, or hair samples. However, such measures do not allow testing for use over previous years.^26^ Moreover, studies with laboratory data and self-reported information have shown that cannabis users reliably report frequency of use and the type of cannabis used.^32^, ^33^

Our potency variable does not include the proportion of another important cannabinoid, cannabidiol (CBD),^34^ because reliable data on this were available for only England and Holland.^17^, ^19^, ^24^, ^25^, ^34^ We categorised the reported types of cannabis used as low and high potency on the basis of the available estimates of mean percentage of THC from official sources. Although this approach does not account for variations in the THC content in individual samples, we used a conservative cutoff of 10%. Given the much higher mean percentage of THC expected in types of cannabis commonly used in UK^24^, ^29^ and in Holland,^19^ our dichotomous categorisation might have led to underestimation of the effect of potency on the ORs for psychotic disorder. Furthermore, a direct measure of the THC content of the cannabis samples used by our participants would have only provided data on THC value for a single timepoint rather than an estimate covering lifetime use.

When setting quotas based on the main sociodemographics of the populations at risk for the recruitment of controls, we applied weights to account for undersampling or oversampling of some groups. For instance, most of the sites oversampled the age group 16–24 years (appendix), which represents the part of the population most likely to consume cannabis^17^ and the most likely to suffer associated harm.^6^, ^16^, ^35^

Moreover, none of the sites mentioned either cannabis, or other, drug use in the materials used for participant recruitment, thus avoiding selection and recall bias. First-episode studies minimise the effect of recall bias, which can be a source of error when history of exposure to environmental factors is collected retrospectively in patients with well established psychosis. This study design also reduces the chances of results being biased by illness course; therefore, it is preferred to investigate aetiology.^36^

In conclusion, our findings confirm previous evidence of the harmful effect on mental health of daily use of cannabis, especially of high-potency types. Importantly, they indicate for the first time how cannabis use affects the incidence of psychotic disorder. Therefore, it is of public health importance to acknowledge alongside the potential medicinal properties of some cannabis constituents the potential adverse effects that are associated with daily cannabis use, especially of high-potency varieties.
