# Supplementary material for: The contribution of cannabis use to variation in the incidence of psychotic disorder across Europe (EU-GEI): a multicentre case-control study
Source: Lancet Psychiatry. 2019 May;6(5):427–36. doi: 10.1016/S2215-0366(19)30048-3 (PMC7646282; doi:10.1016/S2215-0366(19)30048-3)
Supplement: Supplementary appendix [file mmc1.pdf]

THE LANCET
Psychiatry

Supplementary appendix

This appendix formed part of the original submission and has been peer reviewed. We post it as supplied by the authors.

Supplement to: Di Forti M, Quattrone D, Freeman TP, et al, and the EU-GEI WP2 Group. The contribution of cannabis use to variation in the incidence of psychotic disorder across Europe (EU-GEI): a multicentre case-control study. *Lancet Psychiatry* 2019; published online March 19. http://dx.doi.org/10.1016/S2215-0366(19)30048-3.

Appendix:

Table of contents

Methods

Cases and controls recruitment	page 2–6
Incidence rates	page 6
Measures (cannabis use variables)	page 7–8

Statistical analysis

Sensitivity analysis	page 9
Confounders selection	page 10

**References**	page 10–11

s-Table 1. Number of participants of the case-control study recruited by each site who met the inclusion criteria.

s-Table 2. χ^2 and p values for comparisons between those cases who participated in the case-control arm of the study and those who did not. The table shows how the case-control study cases are representative of the rest of the incidence sample by site. (*Age range groups included the following categories: 18–24; 25–34; 35–44; 45–54; 55–64*) (modified from ref 3)

s-Table 3. Representativeness of the control sample compared with the population-at-risk *(This does not include Paris- Maison Blanche where no controls were recruited)(2*)

s-Table 4. Measures of cannabis use included in the analyses

s-Table 5a. Probabilistic sensitivity analysis for selection bias of data on *daily cannabis use*

s-Table 5b. Probabilistic sensitivity analysis for selection bias of data on *high potency cannabis use*

s-Figure 1. FEPp recruitment flow chart

This supplementary material has been provided by the authors to give readers additional information about their work.

Methods

Recruitment:

Cases: We followed procedures previously used to generate representative samples of first episode psychosis patients (FEPp) (1). We identified all individuals aged 18 to 64 years, who contacted mental health services for a suspected first episode of psychosis (FEP), over periods up to four years in 17 catchment areas in England (Southeast London, Cambridgeshire & Peterborough); France (20th arrondissement of Paris, Val-de-Marne, Puy-de-Dôme); the Netherlands (Central Amsterdam, Gouda & Voorhout); Italy (part of the Veneto region, Bologna, and Palermo); Spain (Madrid-Vallecas, Barcelona, Valencia, Oviedo, Santiago, Cuenca), and; Brazil (Ribeirão Preto, Sao Paulo) (full details of the incidence sample recruitment and general description of the incidence study methods are available from the recently published paper by Jogsma et al 2008 (2).

Case ascertainment involved trained researchers making regular contact with all secondary and tertiary mental healthcare providers to identify potential cases and searching electronic clinical records, where available. In this process, all cases with psychosis within services were considered. In all countries, it was uncommon for people to be treated for FEP in primary care; instead people with suspected psychosis would typically be referred to specialist mental health services. Research teams were overseen by a psychiatrist with experience in epidemiological research, and included trained research nurses and clinical psychologists. Teams received training in epidemiological principles and incidence study design to minimize non-differential ascertainment bias across different local and national healthcare systems (see training package on the study website:
(https://www.kcl.ac.uk/ioppn/depts/hspr/research/social-epidemiology-research-group/current-projects.aspx).
As explained in the main text, between May 1, 2010, and April, 1 2015, we approached 1519 patients with first-episode psychosis. Of these 356 (1%) refused to participate, 19 (23%) could not consent because of language barriers and 14 (0·9%) were later excluded (London N=3; Madrid N=2; Bologna N=1; Ribeirão Preto N=8) as they did not meet the age inclusion criteria. For all patients who were not part of the study, local research ethics committees approved the extraction of demographics and clinical information from patient records. Patients who refused to participate were older [$FEP_{consented}$ mean age=30·8 (10.5), median=29.0 (22.0 to 37.0); $FEP_{refused}$ mean age=32·8 (11·5), median=31.0 (25.0 to 42.0); p=0·0015], more likely to be women [$FEP_{consented}$ male=558 (61·9%); $FEP_{refused}$ male=311 (54·7%), $\chi^2(1)=7·6$; p=0·0063] and of White European origin [$\chi^2(5)=38$, p<0·0001] (s-Table2 for details by site). 1130 First Episode Psychosis Patients (FEPp) across the study sites consented to take part in the case-control study (s-**Table 1)**. The FEPp recruited in the case-control study are broadly representative for gender and ethnicity of the rest of the incidence sample. However, in London, Amsterdam and Ribeirao Preto cases aged 18–24 were over-represented in the case-control sample and those aged 45–54 and 55 or over were under-represented compared with the incidence sample (s-**Table 2**)

Supplementary **Table 1**: Number of participants of the case-control study recruited by each site who met the inclusion criteria.

Catchment area		
**England**	**Controls**	**Cases**
Southeast London	230	201
Cambridgeshire	108	45
**The Netherlands**		
Amsterdam	101	96
Gouda & Voorhout	109	100
**Spain**		
Madrid	38	39
Barcelona	37	31
Valencia*	32	49
Oviedo*	39	39
Santiago*	38	28
Cuenca*	38	18
**France**		
Paris (Maison-Blanche)*	0	36
Paris	100	54
Puy-de-Dome	47	15
**Italy**		
Bologna	65	70
Verona*	115	59
Palermo	100	58
**Brazil**		
Ribeiãro Preto	302	192
**Total**	**1,499**	**1,130**

*Sites excluded for the case-control analysis because of missing data ≥10%. Mason-Blanche was excluded from the case-control analysis, as they did not recruit any controls.

Supplementary **Table 2** χ^2 and p-values for comparisons between those cases who participated in the case-control arm of the study and those who did not. The table shows how the case-control study cases are representative of the rest of the incidence sample by site. (*Age range groups included the following categories: 18–24; 25–34; 35–44; 45–54; 55–64*) (modified from 3)

	**Age**				**Gender**				**Minority status**			
	Mean,sd; (Median) case-control sample	Mean; (Median) rest of the incidence sample	χ^2 (based on age groups)	p-value	Male %; N case-control sample	Male %; N rest of the incidence sample	χ^2	p-value	%; N minority Case control	%: N minority Rest of the incidence sample	χ^2	p-value
**England**												
**Southeast London**	29·6,9·4 (27)	34·6,11·2 (33)	**31·4**	**<0·0001**	63·2 (127)	51·4 (112)	**5·9**	**0·0151**	70·6 (142)	77·1 (168)	2·2	0·13
**Cambridgeshire**	28·1,7·9 (26)	32·5,12·3 (29)	6·8	0·15	55·6 (25)	57·0 (126)	0·0	0·86	35·6 (16)	41·8 (87)	0·6	0·44
**The Netherlands**												
**Amsterdam**	27·6,8·1 (25)	38·2,12·5 (36)	**50·5**	**<0·001**	74·0 (71)	59·9 (118)	5·6	0·18	70·8 (68)	73·6 (134)	0·2	0·62
**Gouda & Voorhout**	31.7,11·1 (29)	32.5,12·0 (30)	1	0·9	65·0 (65)	54·6 (36)	1·8	0·18	17 (17)	35·4 (23)	**7·2**	**0·0273**
**Spain**												
**Madrid**	33·1,11·1 (33)	33·9,9·6 (30)	2·5	0·64	69·2 (27)	63·3 (31)	0·3	0.56	10·3 (4)	12·5 (2)	0·1	0·8
**Barcelona**	29·4,11·3 (30)	30·7,13·4 (28)	2·5	0·63	74·2 (23)	50·7 (39)	**5**	**0·0253**	20 (6)	22·4 (15)	0·1	0·79
**Valencia**	31·5,11·4 (27)	35·6,10·3 (35·5)	3·3	0·51	61·2 (30)	20·0 (2)	**5·7**	**0·0170**	16·3 (8)	22·2 (2)	0·2	0·67
**Oviedo**	34·7,10·8 (35)	36·0 9·7 (33)	3·4	0·49	51·3 (20)	46·5 (20)	0·2	0·67	20·5 (8)	12·5 (4)	0·8	0·37
**Santiago**	32·1,11·2 (31)	42·9,10·4 (44)	8·7	0·07	64·3 (18)	37·5 (3)	1·8	0·17	0 (0)	0 (0)	n/a	n/a
**Cuenca**	29·2,9·5 (27)	28·3,11·2 (25)	0·7	0·88	77·8 (14)	77·8 (7)	0·0	1·00	16·7 (3)	33·3% (3)	1	0·33
**France**												
**Paris (Mason Blanche)**	31·4,10·2 (30)	34·1,12·1 (31)	2·9	0·56	66·7 (24)	70·2 (59)	0·1	0·69	58·3 (21)	44.0 (65)	**9.9**	**0·0101**
**Paris**	31·3,10·1 (27)	33.6, 11·2 (30)	4·6	0·33	61·1 (33)	48·1 (75)	2·7	0·1	22·2 (12)	67.9(70)	**22.6**	**0·0004**
**Puy-de-Dome**	37·3,13·4 (32)	33·7,12·7 (34)	8·8	0·07	60·1 (9)	70·4 (19)	0·5	0·49	20·0 (3)	n/a	n/a	n/a
**Italy**												
**Bologna**	32·5,9·9 (33)	33·3,10·5 (30)	7·2	0·13	50·0 (35)	53·7 (51)	0·2	0·64	28·6 (20)	29·5 (28)	0·0	0·9
**Veneto**	36·5,10·1 (37)	36·6,12·3 (36·5)	6·9	0·14	55·9 (33)	52·0 (26)	0·2	0·68	16·7 (9)	20 (10)	0·2	0·66
**Palermo**	30·1,8·9 (28)	34·5,10·2 (31)	12·7	0·01	58·6 (34)	54·6 (66)	0·3	0·6	6·9 (4)	14·1 (17)	1·9	0·16
**Brazil**												
**Ribeirão Preto**	32·3,11·2 (30)	35·9,10·6 (35)	**24·1**	**<0·0001**	56·8 (109)	49·1 (161)	2·9	0·09	49·5 (95)	33·7 (90)	**11·5**	**0·0031**

Controls: All sites contributed to the recruitment of 1499 population controls except for Maison Blanche, which consequently was excluded from the case-control analysis (s-**Table1**). Controls were recruited using a mix of random and quota sampling that aimed to obtain samples representative for age, gender and ethnicity of each site population at risk. Nevertheless, controls aged 18–34 were over-sampled and those aged 35 and over were under-sampled (χ^2=212·4, p<0·0001, s-**Table 3**). Differences by gender and ethnicity are also reported in s-**Table 3**. As reported in the main methods section we used inverse probability weights to account for any over and under sampling of controls relative to the populations at risk; we gave each control's data a weight inversely proportional to their probability of selection, on key demographics (age, gender, ethnicity, using census data on relevant populations). The weights were applied in all analyses.

Supplementary **Table 3:** Representativeness of the control sample compared with the population-at-risk *(This does not include Paris- Maison Blanche where no controls were recruited)(2*)

	Population at-risk		Controls			
	n	Percentage	*N*	Percentage	χ^2	p-value
**Age**						
18-24	1,828,075	**14·1**	323	**21·7**	212·4	<0·0001
25-34	3,057,640	23·6	511	34·3		
35-44	3,058,837	23·7	323	**15·6**		
45-54	2,856,614	21·9	253	17·0		
55-64	2,152,499	16·6	172	11·5		
**Gender**						
Male	6,337,783	**49·5**	672	**46·0**	7·1	0·0077
Female	6,464,653	50·5	788	54·0		
**Minority status**						
Majority	9,881,660	**77.2**	1,072	**72·1**	21·7	<0·0001
Minority	2,917,823	22.8	414	27·9		

Final FEPp and Controls sample size: The controls (N=262) and the cases (N=229) from 6 sites, as reported in s-**Table 1** had *missing* data ≥10% on the main measures of cannabis use and/or on one or more of the main confounding variables, and they were excluded from the analysis resulting in a final number of controls N=1237 and in a final number of cases N=901(see flow chart below, main text Figure 1).

FEPp recruitment flow chart:

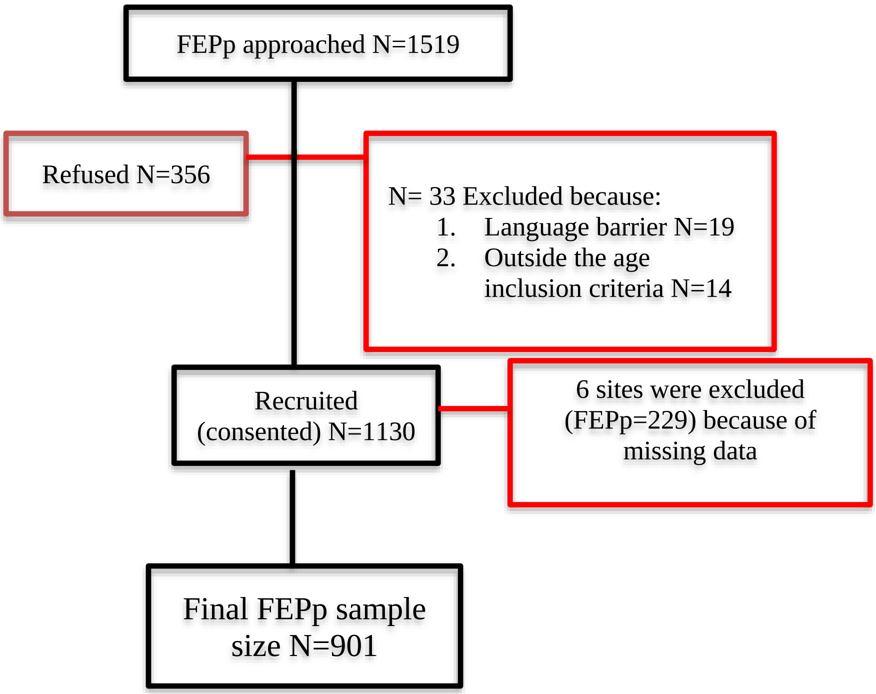

Incidence rates:

The full description of how the Incidence rates for all Psychosis used in the analysis were calculated, can be found in the already published paper by Jongsma et al, 2018 (2). In summary, where case ascertainment is complete and denominator data on the population at risk is available, it is possible to derive estimates of incidence, on the assumption that the population is in a 'steady state' (i.e., the size of the population remains steady over time, even while some individuals leave and some arrive) (2–4). We identified all cases with psychosis in each catchment area and, to determine the denominator, we used country census data for each catchment area (ie, to determine population at risk in each catchment area). With this information, we were able to estimate incidence rates. Puy-de-Dôme (France), data on minority status was missing from the incidence cases for 66% (n=27); therefore, the adjusted IR for this site were not calculated (2), and thus not included in the analysis presented in the graph.

Measures:

The Cannabis Experienced Questionnaire firstly described by Barkus et al 2006 (5), was later modified (CEQmv) (6) to expand 1) questions on the pattern of use including the assessment of the type of cannabis, 2) the section on other drug use and 3) to reduce the section on the experiences following a factor analysis (6). For the EUGEI study we further modified it (CEQ_{EUGEI}) to 1) include questions to assess dependence for cannabis use and other drugs, and 2) to describe use and changes in cannabis use over 3 age periods: 0–11 years old; 12–17 years old and 18 and older.

The Cannabis Experience Questionnaire (CEQ)'s questions we selected to construct our measures of cannabis exposure aimed to ascertain the pattern of use that described the " most" each participant used over the period they used;, thus these were mostly questions covering life-time use rather than current use. : 1) lifetime cannabis use: have you ever used cannabis yes/no; 2) current use: are you currently using cannabis?; 3) age at first use of cannabis in years that in accordance with the existing literature (7) is dichotomized as in s-**Table 4**; 4) frequency of use: "describe how often from the following options": a) I used it only once or twice; b) about once a year; c) few times a year; d) about one/twice a month; e) about once a week; f) more than once a week; g) every day.

5) What type of cannabis did you mostly use? (name given in native language; see next paragraph for more details.

6) How much money did you spent per week ? Choose from: a) less than 2·50 EURO; b) 2·50 to 5·00 EURO; c) 6·00 to 10·00 EURO; d) 11·00 to 15·00 EURO; e) 16·00 to 20·00 EURO; f) above 20·00 EURO. (s-**Table 4).**

Adjusted logistic regressions for age gender and ethnicity were run using the above raw variables as predictors of case-control status. Then for each variable we grouped the listed categories according to the effect size (OR) for case-control status. For instance, the adjusted logistic regression indicated that when using the above raw frequency variables, only the categories "more than once a week" (OR=2·2; 95% CI 1·6 to 2·9) and "everyday" (OR=6·2; 95% CI 4·8 to 8·0) gave ORs significantly greater than 1 for Psychotic Disorders; therefore the categories of frequency variable used in the paper analysis were grouped as follows: a) used never or occasionally (less than once a week); b) used more than once a week (but less than daily); c) used daily .

Supplementary Table 4 : Measures of cannabis use included in the analyses

**Lifetime cannabis use**	**0=never used**	**1=Yes**	
**Currently using cannabis**	0=no use at the time of recruitment in the study and over the previous 4 weeks	1=Yes	
**Age at 1st use of cannabis**	0=never used	1= started at age 16years or older	2=started at age 15 years or younger
**Lifetime frequency of use**	0=used never or occasionally (less than once a week)	1=used more than once a week (but less than daily)	2=used daily
**Money spent weekly on cannabis**	0=never used or spent 20 EURO or less per week	1= spent more than 20 EURO per week	
**Type of cannabis used**	0= never used	1= used types with THC<10%	2=used types with THC=>10%

The cannabis potency variable:

The potency variable was created using a cut off of THC=10% based on the mean THC concentration expected in the different types of cannabis available across the side sites, as reported in the EMCDDA and by the National data on cannabis potency quoted (8). Participants were asked to name in their own language the name of the type of cannabis they mostly used during their period of use.

The low-potency cannabis category (THC<10%) included hash/resin from UK and Italy, imported herbal cannabis from UK, Italy, Spain and France, Brazilian marijuana and hash and the Dutch Geimporteerde Wiet. The high-potency category (THC=>10%) included all the other types reported by the study participants in their original language street names such as: UK home-grown skunk/sensimilla UK Super Skunk, Italian home-grown skunk/sensimilla , Italian Super Skunk, the Dutch Nederwiet, Nederhasj and geimporteerde hasj, the Spanish and French Hashish (from Morocco),

Spanish home-grown sensimilla, French home-grown skunk/sensimilla/super-skunk and Brazilian skunk (9–16).

Statistical analysis:

Selection bias:

We ran a probabilistic sensitivity analysis to estimate the potential impact of selection bias, using the *episensi* commands in Stata. This involves: 1) selecting a random sample (one set of bias parameters) from the specified probability density functions of the bias parameters [e.g. Selection bias factor: Log-Normal (0.00, 0.21)], and 2) calculating a bias-corrected OR from the selected parameters. Both steps are repeated many times (we ran repetitions=20000) to obtain a distribution of bias-corrected ORs (ref 27 main text).

Table 5a reports the original OR (conventional estimate) and the corrected one (systematic and random error estimate) in the 50-percentile column, within the corresponding 95% CI values. The selection-bias corrected OR (OR=5·7, 95% CI 3·5 to 9·4) for daily cannabis use compared to the original OR (OR=5·7, 95% CI 4·4 to 7·5) (s-Table 5a) was barely changed. However, the confidence limits were wider, suggesting a wider range of possible values for the true OR with 95% certainty. We found a similar pattern of results for the probabilistic sensitivity analysis to estimate the potential effects of selection bias of data on high potency cannabis use as shown in table (s-Table 5b). Both set of analyses suggest that selection bias is unlikely to explain our findings.

Supplementary Table **5 a**: Probabilistic sensitivity analysis for selection bias of data on *daily cannabis use* assuming lognormal distribution with mean 0 and standard deviation 0.21 [Selection bias factor: Log-Normal (0.00, 0.21)], number of repetitions=20000 and seeds=123.

	**Percentiles**			**Ratio**
	**2.5**	**50**	**97.5**	**97.5/2.5**
**Conventional**	4.4	5.7	7.5	1.7
**Systematic error**	3.8	5.7	8.6	2.3
**Systematic and random error**	3.5	5.7	9.4	2.7

Supplementary Table **5 b**: Probabilistic sensitivity analysis for selection bias of data on use of *high potency cannabis* assuming lognormal distribution with mean 0 and standard deviation 0.21 [Selection bias factor: Log-Normal (0.00, 0.21)], number of repetitions=20000 and seeds=123.

	**Percentiles**			**Ratio**
	**2.5**	**50**	**97.5**	**97.5/2.5**
**Conventional**	1.9	2.3	2.8	1.5
**Systematic error**	1.5	2.3	3.5	2.3
**Systematic and random error**	1. 5	2.3	3.7	2.5

Confounder selection: we tested for an association between the available a) socio-demographic data and b) data on drug use, with case-control status in the whole sample. All the socio-demographic variables available and in line with the existing literature[1] were associated with case-control status.

Only the variables on drug use associated with case-control status are reported in **Table 2** (eg, data on Alcohol use are not in the table).

To estimate the possible confounding effect of tobacco smoking in our analysis, we used the data on number of cigarettes smoked over the past 12 months. As for the method used to group the raw measures of cannabis exposure, we applied a logistic regression adjusted for age, gender and ethnicity, testing for an association between the raw variable on number of cigarettes smoked per day over the previous 12 months (0=never smoked; 1=smoked less than cigarettes per day; 2= smoked 10 or more cigarettes) and case-control status. Smoking less than 10 cigarettes per day was not associated with an increase in the ORs for psychotic disorder (OR=0·9; 95% CI 0·9 to 2·8) compared to never smoked, contrary to smoking 10 cigarettes or more (OR= 2·5 95% CI 1·7 to 4·2). Therefore, the variable on tobacco use entered in the main analysis model is the one described in **Table 2**.

To test if alcohol use was associated with case-control status we used the following data-collected: 1) life-time alcohol use (yes/no); 2) "did you drink at least 12 or more alcoholic beverages in the past 12 months? (yes/no); 3) How many drinks did you drink every day on an average week?

In the whole sample analysis (FEPp=901; Controls=1237), none of these measures of alcohol consumption were associated with being a case (FEPp). On the contrary, 75% (N=927) of controls compared to 63% (N= 567) of FEPp reported having drunk an alcoholic beverage at least once in their life-time (χ^2=27·9; p=0.001). Moreover, 61% (N= 754) of controls compared to 40% (N= 360) of cases reported having drunk 12 or more alcoholic beverages in the past 12 months. Also, we found no difference between cases and controls in the mean number of alcoholic drinks every day on an average week [Controls: mean=5·2 (0·4), median=2.0 (0.0 to 6.0); FEPs: mean=4·8 (0.4), median=1.0 (0.0 to 4.0); t=0·8; df=2136; p=0·45].

Moreover, adding, the above measures of alcohol consumption to the multivariable logistic regression did not confound the tested association between cannabis use and psychotic disorder.

Supplementary methods reference:

1. Fearon P, Kirkbride JB, Morgan C, Dazzan P, Morgan K, Lloyd T, et al. Incidence of schizophrenia and other psychoses in ethnic minority groups: results from the MRC AESOP Study. Psychological Medicine. 2006; 36(11):1541-50.

2. Jongsma HE, Gayer-Anderson C, Lasalvia A, et al. Treated incidence of psychotic disorders in the multinational eu-gei study. JAMA Psychiatry. 2018; 75(1):36-46.

3. Jongsma HE. The role of the sociocultural context in explaining variance in incidence of psychosis and higher rates of disorder in minorities. [PhD Thesis]: University of Cambridge; 2018.

4. Vandenbroucke JP, Pearce N. Case–control studies: basic concepts. International Journal of Epidemiology. 2012; 41(5):1480-9.

5. Barkus EJ, Stirling J, Hopkins RS, Lewis S. Cannabis-induced psychosis-like experiences are associated with high schizotypy. Psychopathology. 2006; 39(4):175-8.

6. Di Forti M, Morgan C, Dazzan P, Pariante C, Mondelli V, Marques TR, et al. High-potency cannabis and the risk of psychosis. British Journal of Psychiatry. 2009; 195(6):488-91.

7. Casadio P, Fernandes C, Murray RM, Di Forti M. Cannabis use in young people: The risk for schizophrenia. Neuroscience & Biobehavioral Reviews. 2011; 35(8):1779-87.

8. European Monitoring Centre for Drugs and Drug Addiction (EMCDDA). European Drug Report 2016: Trends and Developments. Publications Office of the European Union. Luxembourg 2016.

9. European Monitoring Centre for Drugs and Drug Addiction (EMCDDA), Ministry of Health and Consumer Affairs (Spain). Spain National Report to the EMCDDA 2012.

10. Niesink R RS. THC-concentraties in wiet, nederwiet en hasj in Nederlandse coffeeshops (2012-2013). AF1221. Utrecht: Trimbos-instituut; 2013.

11. Observatoire Français des Drogues et des Toxicomanies (OFDT). Drogues, chiffres clés. Paris 2015.

12. Zamengo L, Frison G, Bettin C, Sciarrone R. Cannabis potency in the Venice area (Italy): Update 2013. Drug Testing and Analysis. 2015; 7(3):255-8.

13. Niesink RJM, Rigter S, Koeter MW, Brunt TM. Potency trends of Δ9-tetrahydrocannabinol, cannabidiol and cannabinol in cannabis in the Netherlands: 2005–15. Addiction. 2015; 110(12):1941-50.

14. de Oliveira GL, Voloch MH, Sztulman GB, Neto ON, Yonamine M. Cannabinoid contents in cannabis products seized in São Paulo, Brazil, 2006–2007. Forensic Toxicology. 2008; 26(1):31-5.

15. Potter DJ, Clark P, Brown MB. Potency of Δ9–THC and Other Cannabinoids in Cannabis in England in 2005: Implications for Psychoactivity and Pharmacology*. Journal of Forensic Sciences. 2008; 53(1):90-4.

16. Hardwick S KS. Home Office Cannabis Potency Study 2008. London: Home Office Scientific Development Branch; 2008.
